# Comparison of retropubic tension-free vaginal tape inserted on two different height positions

**DOI:** 10.1007/s00192-021-05056-7

**Published:** 2022-01-17

**Authors:** Anna Pawlaczyk, Piotr Wąż, Marcin Matuszewski

**Affiliations:** 1 Department of Urology, Medical University of Gdansk, ul. Smoluchowskiego 17, 80-214 Gdańsk, Poland; 2 Department of Nuclear Medicine, Medical University of Gdansk, Gdansk, Poland

**Keywords:** Midurethral tape, TVT, Surgery, Ultrasonography, Stress urinary incontinence

## Abstract

**Introduction and hypothesis:**

Surgical treatment using the mid-urethral tape has become a gold standard in the treatment of stress urinary incontinence in women. Many urogynecologists use ultrasound during the postoperative follow-up. The aim of this study was to investigate whether the position of the tape in the mid- or distal-urethra could influence the subjective assessment after surgery in 1-month control based on questionnaires of genitourinary symptoms, UDI6-SF and VAS scale.

**Methods:**

A group of 76 patients using a synthetic tension-free retropubic vaginal tape after anti-incontinence surgery was retrospectively included in this study. In a postoperative follow-up, the synthetic tape detection was performed using introital ultrasound, and its position was determined as a quotient T/U (T = distance between the external urethral orifice and the lower edge of the tape, U = urethral length). The patients were divided into two groups of 38 patients: one group with the position of the tape in the distal urethra (T/U ≤ 0.24) and the other group with the tape localised in the mid-urethra (T/U = 0.25–0.37). The correlation between the height of the tape position and the subjective assessment was evaluated in both groups of patients in the 1-month control.

**Results:**

No association was found between the height of the tape position in a group of patients after anti-incontinence surgery with a T/U value not exceeding 0.375 and the subjective assessment or the value of Vres.

**Conclusions:**

The height of the tape position, with the T/U not exceeding 0.375, has no impact on the subjective assessment of the surgical anti-incontinence treatment in 1-month control.

## Introduction

Surgical treatment of stress urinary incontinence in women using a polypropylene tape inserted without tension under the mid-urethra has been one of the most commonly employed and extensively studied surgeries from its introduction in 1995 till now [1, 2]. This minimally invasive procedure, described as ambulatory, is very effective with cure rates up to 93% at 6 months and 86% at 3 years [3, 4]. Despite all efforts, a small group of patients remain dissatisfied with the treatment. Numerous studies are constantly ongoing on how to increase effectiveness and avoid failure. According to our previous study, which was carried out in a group of patients dissatisfied with the treatment, the conclusion was set that the tape localisation with the lower edge too high, that is > 37.5% (T/U > 0.375) of the urethral length determined in introital ultrasound, may result in treatment failure [5]. The same observations were described by others [6, 7]. The aim of this retrospective study was to investigate whether the position of the tape in the mid- or distal-urethra, that is, according to our previous study and reports from other investigators to be an optimal place, with the lower edge of the tape < 37.5%, could influence the subjective assessment after surgery in 1-month control.

## Materials and methods

Seventy-six patients treated surgically for stress urinary incontinence between 2015 and 2019 were retrospectively enrolled into the study. All subjects received the same treatment: retropubic tension-free vaginal tape (TVT) using the same type of tape with the same technique of insertion, as described by Ulmsten and Petros [1]. Exclusion criteria were concomitant neurogenic or idiopathic detrusor overactivity and predominant symptoms of overactive bladder (OAB) present before surgery, patients with neurological diseases affecting lower urinary tract function and patients after radiotherapy of the pelvis. Qualification for surgical treatment was based on medical history, urogynecological examination, introital ultrasound of the lower urinary tract with the sonographic assessment of residual urine, completing the Urogenital Distress Inventory (UDI6) short form, visual analogue scale (VAS) of subjective assessment of lower urinary tract function, ranging from 0 to 100 (0 corresponding to very bad assessment and 100 to very good) and a questionnaire of severity of genitourinary symptoms, ranging from 0 to 3 (0 = not at all, 1 = slight, 2 = moderate, 3 = severe complaints) (Table 1).

**Table 1:** Questionnaireon genitourinary symptoms and VAS of subjective assessment of lower urinary tract function (LUT) before and 1 month after surgery

Are you suffering from?	0: not at all	1: slightly	2: moderate	3: severe complaints
Pelvic pain				
Dyspareunia				
Hematuria				
Vaginal discharge				
Frequency				
Nocturia				
Urgency				
Urinary incontinence				
Stress urinary incontinence				
Hesitancy				
Dysuria				

How do you assess the LUT function?

Very bad excellent

Introital ultrasound was performed by an experienced ultrasonographer in the semi-sitting position of the patient, just after emptying the bladder, at rest, using B-K Medical and an endocavity probe with a frequency of 9 MHz. Preoperative visualisation of the lower urinary tract allows to exclude the presence of contraindications to surgical treatment: bladder emptying abnormalities (residual volume after voiding), the presence of previously inserted polypropylene materials, and the presence of urethral diverticula and periurethral cysts. During the introital sonography, we check the condition of the pelvic floor and the patient’s ability to perform the pelvic floor muscle training by themself, which is an important part of the treatment process.

A TVT procedure with a polypropylene monofilament macroporous tape inserted retropubically without tension under the mid-urethra was performed according to the technique described by Ulmsten and Petros [1]. To prevent cephalic displacement of the tape we applied one fast absorbable suture on the lower edge and the periurethral tissue just as others do [7]. For proper intraoperative tape tensioning the straight Pean clamp technique was performed [9]. All patients received antibiotics perioperatively. After the operation, a follow-up control was performed, usually after 1 month.

As a follow-up, apart from the medical history and the introital sonographic localisation of the tape, all subjects completed the same questionnaires as before: the UDI6 short form, the severity of genitourinary symptoms with a scale from 0 to 3 and the VAS subjective assessment of the lower urinary tract function.

**Fig. 1:**
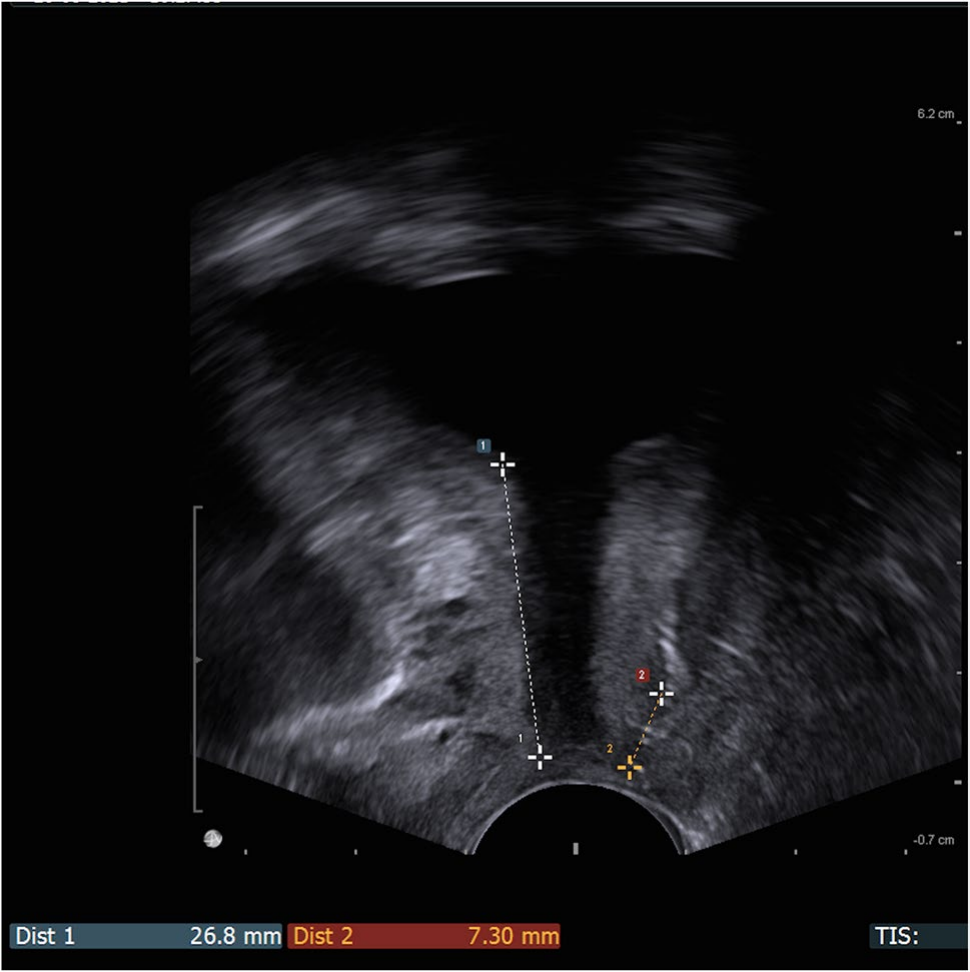
Ultrasonographic tapevisualisation in mid-sagittal scan: Dist 1: urethral length (U), Dist 2: distance from the external urethral orifice to the lower edge of the tape (T)

**Fig. 2:**
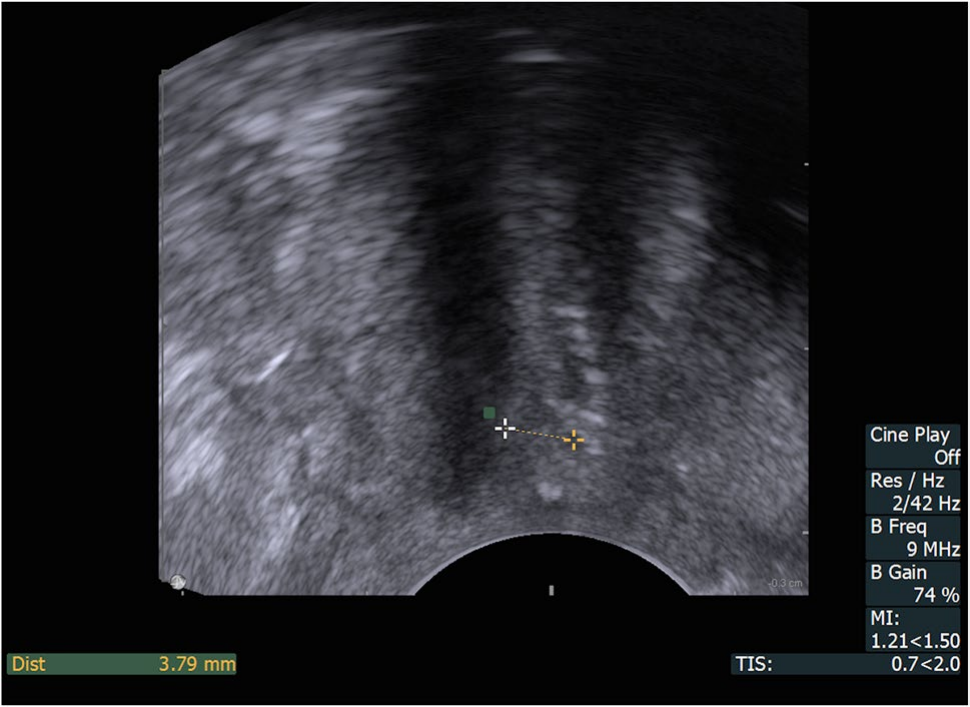
Ultrasonographic tape visualisation in mid-sagittal scan: Dist: distance between the lower edge of the tape and the hypoechogenic urethral complex (T_UC_)

**Fig. 3:**
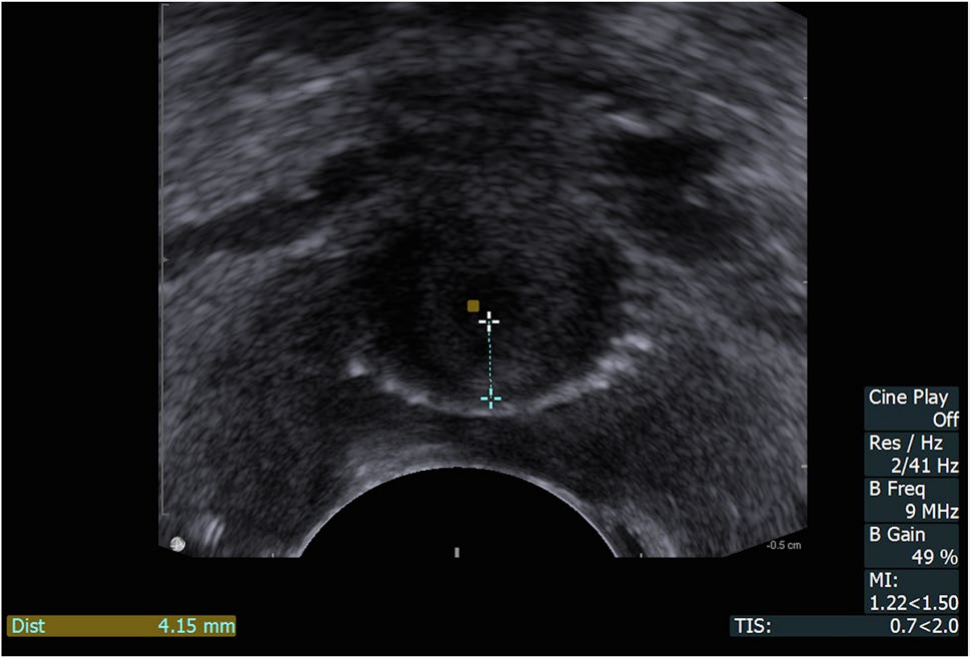
Ultrasonographic tape visualisation in a transverse scan: Dist: distance between the tape and the urethral lumen (T_UL_)

The ultrasound examination was performed immediately after emptying the bladder to allow us to evaluate the residual urine. The technique of the introital sonographic localisation of the tape was detailed in our previous report [5]. During the ultrasound there were four key measurement points assessed, presented in Figs. 1, 2 and 3:

- in mid-sagittal scan:
  - urethral length (U) (Fig. 1)
  - distance between the external urethral orifice and the lower edge of the tape (T) (Fig. 1)
  - distance between the lower edge of the tape and the hypoechogenic urethral complex (UC = longitudinal smooth muscle and urethral vascularisation, T_UC_) (Fig. 2)
- in a transverse scan:
  - the distance between the tape and the urethral lumen (T_UL_) (Fig. 3).

The zero point of the urethral length was assumed to be the external urethral orifice, contrary to the reports of many investigators, because in our study it was the most visible, independent and unchangeable. The urethral internal orifice depicted at anterior urethral wall was assumed to be at 100% of the urethral length. The level of the tape position was assessed as a quotient T/U (T = distance from the external urethral orifice to the lower edge of the tape; U = length of the entire urethra) determined as a percentage of the urethral length. We took the lower edge of the tape as a reference, because it is best seen in introital ultrasound and marks the end of the tape.

The patients were divided into two groups of 38 patients depending on the position of the tape in relation to the percentage of the urethral length: one group with a low position of the tape, in the distal urethra, with the lower edge of the tape at a height of 24% and below, and the other group with the location of the lower edge within 25–37%, in the mid-urethra. The current study did not include patients whose lower edge of the tape was located higher than 37.5% of the urethral length in the postoperative control after 1 month, because in our previous study it was found that these patients were more likely to develop bothersome LUTS [5].

Both groups of patients were homogeneous in terms of age, BMI, the number of TVT procedures performed, a history of vaginal surgeries, the tape complementary surgeries and also the time between the operation and control. In none of the patients, during the secondary surgery with the use of the tape, was there a duplication of the polypropylene material, because in our centre, after a failure, we always remove the malfunctioning tape before the secondary operation (Table 2).

**Table 2:** Characteristics of the groups of patients

Characteristics of the groups of patients					
		T/U value (number of patients) <=0.24 (*n* = 38)	T/U value (number of patients) 0.25–0.37 (*n* = 38)	*p* value	Statistics
T_UL_ mean (min–max)		6.17 (3–9)	6.3 (3–10)		
Age, mean (Min–max) Median		56.66 (32–78) 58	57.13 (36–77) 56.5	0.8485	n.s. *
BMI (body mass index) mean (Min–max) Median		28.79 (21–38) 28.5	27.95 (20–35) 28	0.3724	n.s. *
TAPE = secondary surgery TAPE = primary surgery		1 37	1 37	1	n.s. ***
Number of patients	With previous anterior colporrhaphy Without previous anterior colporrhaphy	2 38	3 35	1	n.s. ***
Number of patients	With additional anterior colporrhaphy Without additional anterior colporrhaphy	1 37	5 33	0.1997	n.s. ***
Number of patients	With additional posterior colporrhaphy Without additional posterior colporrhaphy	2 36	3 35	1	n.s. ***
Time between surgery and follow-up (months) Mean (min–Max) Median		2.24 (1–8) 1	2.64 (1–8) 1	0.4097	n.s. **

Statistical significance level α = 0.05

n.s. = not significant

*Two sample *t*-test

**Wilcoxon-Mann-Whitney test

***Fisher’s exact test for count data

Both groups of patients were compared in terms of subjective assessment of the treatment and other conditions that may affect it by analysing the following:

- whether the position of the tape (higher 25–37%, lower ≤ 24%) affects the subjective assessment of surgical treatment in the control after one month.
- whether the value of T/U correlates with the postoperative value of Vres.
- whether the BMI value could influence the choice of the height of the tape insertion.
- whether the values of T_UC_ and T_UL_ correlate with subjective assessment.

For quantitative variables, basic statistics were calculated, i.e. mean, median, and the minimum and the maximum values. The Shapiro-Wilk test was used to determine whether the values of the analysed variables came from a population with a normal distribution.

The differences between the groups of quantitative variables were tested using the Student’s *t*-test or Wilcoxon rank sum test. The type of the above-mentioned tests (and additional options) was selected depending on the *p* value of the Shapiro-Wilk test and the homogeneity of variance test.

For determining the correlations between the selected groups and the values of quantitative variables, the Pearson’s r, Kendall’s τ and Spearman’s ρ coefficients were calculated. In each of the mentioned cases, it was determined whether the obtained coefficients were statistically significant.

For qualitative variables, the basic statistic was the number of patients belonging to a specific category. The relationship between the categories was tested using the Fisher’s exact test for count data.

A one-dimensional ordinal regression model was created for the UDI6 variable. In the constructed mathematical models, the UDI6 variable was the dependent variable, and the BMI, Vres and T/U variables were independent variables. Four multiple ordinal regression models were also created where the independent variables were BMI and T/U. In the models, the values of the T/U variable were divided into two groups. Four such divisions were made depending on the selected cut-off point (0.2; 0.24; 0.25; 0.26).

For each of the tests used, the significance level was set at α = 0.05. This is a retrospective causal-comparative study. The study was approved by The Independent Ethics Committee at The Medical University of Gdansk, (NKBBN/2/2018).

## Results

The results are presented in Table 3. There was no correlation between the height of the tape position in relation to the urethra in a group of patients with a T/U value not exceeding 0.375 and the subjective assessment. The T/U value up to 0.375 had no correlation with the value of VAS. In patients with higher values of BMI, the probability of obtaining UDI6 = 0 in the T/U > 0.2 group decreased much faster than in the group with T/U < = 0.2 and it was statistically significant.

**Table 3:** Patient characteristics in follow-up after surgery

Patient characteristics in follow-up after surgery				
Analysed data	Results		*P* value	Statistics
	T/U value ≤ 0.24	T/U value 0.25–0.37		
T/U mean Min–max Number of patients	Mean: 0.18 0.09–0.24 (*n* = 38)	Mean: 0.30 0.25–0.37 (*n* = 38)		
UDI6-SF mean (Min–max) Median 1st Qu./3rd Qu.	0.9211 (0–9) 0.0 0.0/0.75	1.158 (0–6) 0.5 0.0/2.0	0.0829	n.s.***
Incontinence (0–3) mean (min–max) Median 1st Qu./3rd Qu.	0.1316 (0–3) 0.0 0.0/0.0	0.07895 (0–1) 0.0 0.0/0.0	0.9822641	n.s.***
Stress urinary incontinence (0–3) mean (min–max) Median 1st Qu./3rd Qu.	0.02632 (0–1) 0.0 0.0/0.0	0.2632 (0–1) 0.0 0.0/0.0	1	n.s.***
VAS (0–100) mean (min–max) Median 1st Qu./3rd Qu.	86 (19–100) 90 81/97	89.63 (44–100) 92 82.25/100	0.2135829	n.s.***
Residual urinae (ml) mean (min–max) Median	6.79 (0–100) 0	10 (0–100) 0	0.6271	n.s.**
Intraoperative complications				
Bladder injury			0.1075	n.s.****
Number of patients with bladder injury	1	6		
Number of patients without bladder injury	37	32		
%	2.6%	15.8%		
Blood loss (ml) mean (min–max) Median	62.24 (0–500) 50	54.47 (5–300) 40	0.598	n.s.**

Statistical significance level α = 0.05

n.s. = not significant

1st Qu, 3rd Qu = 1st quartile, 3rd quartile

**Wilcoxon-Mann-Whitney test

***Wilcoxon rank sum test with continuity correction

****Fisher’s exact test for count data

The tape inserted in higher position (T/U 0.25–0.375) was associated with intraoperative bladder injury more often than the tape inserted in lower position (T/U 0.09–0.24), but this was not statistically significant.

The values of T_UC_ and T_UL_ did not influence the subjective assessment of surgical treatment in 1-month control.

## Discussion

Surgical treatment using mid-urethral polypropylene tape has become a gold standard in the treatment of stress urinary incontinence in women and is also the best-studied surgical technique for this indication. Many urogynecologists use ultrasound in postoperative follow-up [3, 5–16]. There are different techniques of ultrasound examination described in the literature: imaging with a curved linear array scanner in perineal ultrasound, with a transvaginal linear array transducer in 3D imaging, and transrectal or introital sonography. Our centre uses introital sonography. Ultrasound allows for the best visualisation of the location of the implant, its behaviour in dynamic evaluation during increased intra-abdominal pressure and possible migration to adjacent organs. To accurately determine the position of the tape in relation to the urethra, investigators use the position of the lower edge of the tape or the midpoint as a reference point, determining the position of this point in relation to the total urethral length as a percentage [5, 6, 10, 14]. Some authors consider the bladder neck to be 0%, others the external orifice of the urethra [5, 6]. Regardless of the definitions of the tape location in relation to the urethra, many studies show that the tape located in the proximal part of the urethra promotes the development of LUTS and OAB or recurrent SUI [5–7, 14]. In our previous study we analysed the tape position in a group of patients suffering from bothersome LUTS showing that the tape was located in the proximal part of the urethra with the lower edge of the tape above 37.5% of the urethral length [5].

In the present study, we assessed the influence of the position of the tape (from 9 to 37.5% of the length of the urethra) on the treatment results. We found no differences in subjective assessment among patients with tape located at different heights ranging from 9 to 37.5% of the urethral length in 1 – month control.

Kociszewski stated that the optimal zone for locating the tape is 50–80% of the urethral length in a 6-month follow-up with the tape midpoint as a reference. In his works, he showed that the failure was related to the location of the tape in the proximal part of the urethra but he did not state that the tape location in the distal part of the urethra would be associated with failure [6]. After analysing his conclusions with our results, it turns out that the conclusions are the same.

Duckett pointed out that there was a significant reduction in maximal flow rate (MFR) if the tape was positioned under the mid-urethra, while the tape positioned under the distal part of the urethra had no effect on the MFR value at postoperative follow-up. He also observed a trend towards the increase in detrusor pressure at maximum flow in pressure flow studies in patients with mid-urethral tape compared to patients with distal-urethral tape. The conclusion was that the distal-urethral tape would be less obstructive [15].

Probably a better subjective assessment consists not only of the relief of symptoms of stress urinary incontinence but also more comfortable emptying of the bladder. Also, among patients with high BMI, the location of the tape below 20% of the urethral length was associated with a higher probability of the better assessment in UDI6.

In our retrospective study we found a significant impact of the value of residual urine volume (Vres) determined by ultrasound in 1-month follow-up on the subjective assessment of surgical treatment.

Bogusiewicz, in the ultrasound examination of the tape position after surgery, found that the tape, which was located both above the optimal zone (the middle point of the tape at 50–75% of the urethral length) and below, was associated with treatment failure. In the subsequent study, he analysed the position of the tape in ultrasound control on the day of discharge and stated that the tape could run in the middle part or distal part of the urethra. Proximal placing could lead to treatment failure [7, 16].

Limitations of our study are the small patient population and the short-term follow-up after surgery. In long-term follow-up, we expected too many different factors influencing the effectiveness of the treatment, so we abandoned long-term follow-up. The limitations also include the comparison of the tape inserted retropubically with the tape inserted transobturatory. The last technique dominates in the latest available literature.

## Conclusion

The retropubical tape insertion, regardless of whether it is located in the middle or distal part of the urethra, does not affect the subjective assessment of surgical treatment or the residual volume in the 1-month postoperative follow-up. Inserting the tape higher could be associated with higher risk of bladder injury, but this was not statistically significant. Disordered bladder emptying has a negative impact on subjective assessment. Among patients with higher BMI values, the tape inserted at the height and < 20% of the urethral length could be better in the subjective assessment. The values obtained by ultrasound of the distance of the tape from the hypoechoic structures (T_UC_) of the urethra in the mid-sagittal view and transverse scan (T_UL_) are not related to the subjective assessment of the treatment.

## Declarations

### Conference presentations

Prior study “Variations in midurethral tape localization and early outcomes—a preliminary report” was presented at the ICS Annual Meeting 2018 in Philadelphia, PA.
